# Case Report: *FOXP3* Mutation in a Patient Presenting With ALPS

**DOI:** 10.3389/fimmu.2021.692107

**Published:** 2021-08-31

**Authors:** Afef Rais, Najla Mekki, Faten Fedhila, Mohammed Faraj Alosaimi, Monia Ben Khaled, Amal Zameli, Nourhen Agrebi, Maryam Kallel Sellami, Raif Geha, Imen Ben-Mustapha, Mohamed-Ridha Barbouche

**Affiliations:** ^1^Laboratory of Transmission, Control and Immunobiology of Infections (LR11IPT02), Institut Pasteur de Tunis, Tunis, Tunisia; ^2^Faculty of Medicine, Université de Tunis El Manar, Tunis, Tunisia; ^3^Université de Tunis El Manar, Tunis, Tunisia; ^4^Department of Pediatrics A, Children’s Hospital, Tunis, Tunisia; ^5^Department of Pediatrics, College of Medicine, King Saud University, Riyadh, Saudi Arabia; ^6^Pediatric Immuno-Hematology unit, Bone Marrow Transplantation Center Tunis, Tunis, Tunisia; ^7^Department of Immunology, La Rabta University Hospital, Tunis, Tunisia; ^8^Division of Immunology, Boston Children’s Hospital, Harvard Medical School, Boston, MA, United States

**Keywords:** FOXP3, IPEX, ALPS, NGS, inborn errors of immunity

## Abstract

ALPS and IPEX are two well-characterized inborn errors of immunity with immune dysregulation, considered as two master models of monogenic auto-immune diseases. Thus, with autoimmunity as their primary clinical manifestation, these two entities may show clinical overlap. Traditionally, immunological biomarkers are used to establish an accurate differential diagnosis. Herein, we describe a patient who presented with clinical features and biomarkers fulfilling the diagnostic criteria of ALPS. Severe apoptotic defect was also shown in the patient’s cell lines and PHA-activated peripheral blood lymphocytes. Sanger sequencing of the *FAS* gene did not reveal any causal mutation. NGS screening revealed a novel deleterious variant located in the N terminal repressor domain of *FOXP3* but no mutations in the FAS pathway-related genes. TEMRA cells (terminally differentiated effector memory cells re-expressing CD45RA) and PD1 expression were increased arguing in favor of T-cell exhaustion, which could be induced by unrestrained activation of T effector cells because of Treg deficiency. Moreover, defective FOXP3 observed in the patient could intrinsically induce increased proliferation and resistance to apoptosis in T effector cells. This observation expands the spectrum of FOXP3 deficiency and underscores the role of NGS in detecting mutations that induce overlapping phenotypes among inborn errors of immunity with immune dysregulation. In addition, these findings suggest a potential link between FOXP3 and FAS pathways.

## Introduction

Primary immune dysregulation diseases are a group of monogenic disorders characterized by defective immune regulatory pathways (1–3). Their clinical presentation mainly comprises organ-specific autoimmunity, hyperinflammation, and nonmalignant lymphoproliferation. New sequencing technologies have improved the understanding of this ever-expanding group of disorders but they still often pose a diagnostic challenge because of their variable and complex phenotypic expressions and the overlap of symptoms between different entities (1, 4). Immunodysregulation, polyendocrinopathy, enteropathy, X-linked (IPEX) and autoimmune lymphoproliferative syndrome (ALPS) are archetypes of inborn errors of immunity (IEI) with immune dysregulation and have gained attention as models of monogenic autoimmunity.

IPEX syndrome is secondary to mutations in the Forkhead Box Protein 3 (FOXP3) gene, a transcription factor essential for regulatory T cell differentiation and function. Its clinical expression encompasses various combinations of autoimmune manifestations appearing early in life, with a characteristic triad comprising enteropathy, dermatitis, and autoimmune endocrinopathy, usually, type 1 diabetes (5).

ALPS represents a distinct pathologic mechanism for loss of immune tolerance caused by a defect in the Fas-FasL pathway. Noninfectious and nonmalignant lymphoproliferation associated with autoimmune cytopenias and a greatly increased lifetime risk of lymphoma constitute the major features of ALPS. The marking immunological phenotype remains an expansion of autoreactive double-negative T (DNT) cells (6), a subset of CD3+ cells bearing α/β T cell receptor (TCR), and negative for both CD4 and CD8 co-receptors. They massively accumulate in patients with ALPS but are also reported in other inflammatory and autoimmune conditions (7, 8). A chronic non-malignant lymphadenopathy, an elevation of peripheral DNT cells, and a defective apoptosis assay were first proposed as a required triad of criteria to establish the diagnosis of ALPS (9). Later on, serum sFasL, IL-10, and vitamin B12 were shown to be elevated in ALPS because of *FAS* mutations (ALPS-FAS), and their association to high DNT cells rose as an accurate predictor for the presence of germline or somatic *FAS* mutation (10, 11). Consequently, these biomarkers were included in the revised diagnostic criteria of ALPS to facilitate the patients’ identification particularly in settings where no access to advanced genetic analysis or functional testing are available (12).

The functional apoptosis test allows to detect a defective response upon Fas stimulation which results in abnormal cell survival and is sufficient for definitive ALPS diagnosis provided that required criteria are fulfilled (12). Therefore, defective Fas driven apoptosis is considered specific to ALPS and prompts molecular testing when a patient has suggestive clinical and laboratory features.

Despite the establishment of well-characterized ALPS diagnostic guidelines, phenotypic overlap with other primary immune dysregulation conditions still poses a diagnostic challenge (13). Indeed, previous reports have shown that LRBA deficiency, *STAT3* GOF mutations and ITK deficiency may be misdiagnosed as ALPS and may share with it some clinical and immunological features (13–17).

Herein, we describe a patient bearing a novel *FOXP3* mutation in the N terminal repressor domain of the protein who fulfills the current criteria for definitive diagnosis for ALPS (12, 18).

## Case Description

**Figure 1:**
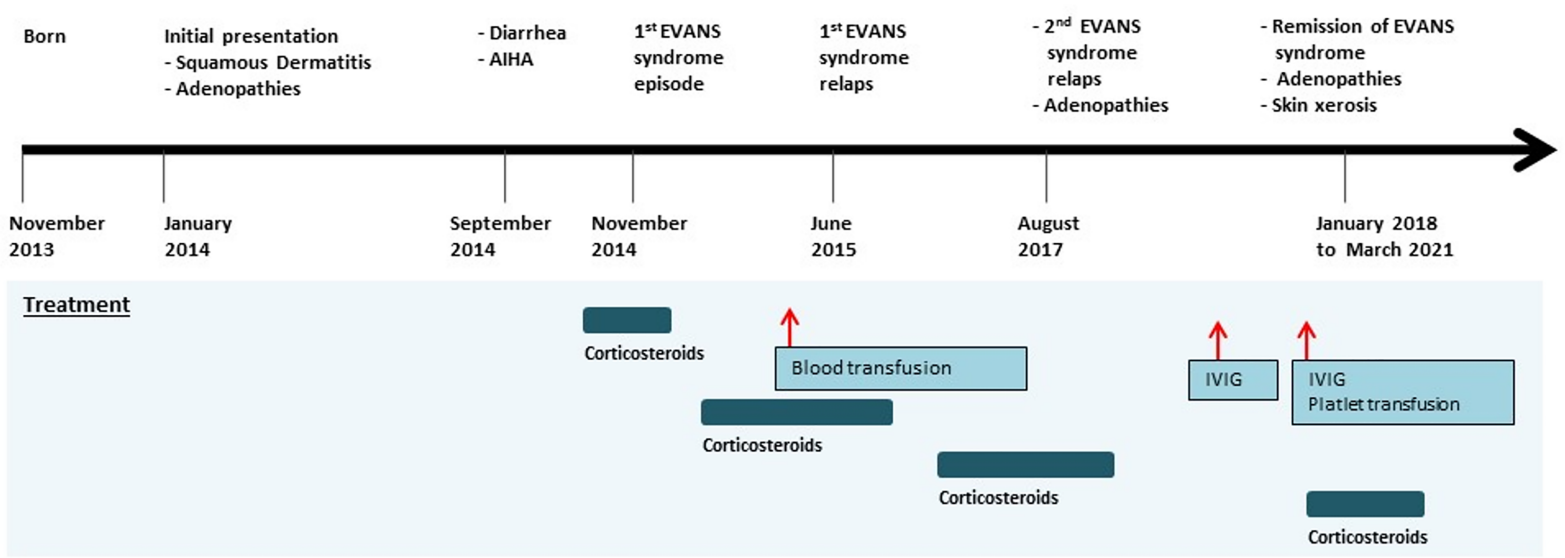
Timeline of the clinical events and treatment strategies. AIHA, autoimmune hemolytic anemia; IVIG, intravenous immunoglobulin.

Born to a non-consanguineous marriage, the patient had a family history of infant deaths without clearly identified causes. His clinical manifestations started at the age of 2 months and were marked by generalized squamous dermatitis and multiple adenopathies without hepatosplenomegaly. Langerhans cell histiocytosis diagnosis was initially proposed based on the presence of multiple CD1a+PS100+ cells in a retroauricular lymph node biopsy, then ruled out because of the atypical cutaneous manifestation and the absence of histiocytic proliferation. The dermatitis significantly improved after the use of topical corticosteroids and evolved to skin xerosis. At the age of 10 months, the clinical course was marked by the occurrence of a severe prolonged diarrhea episode with edema and hypoproteinemia requiring hospitalization. Etiological investigations, including coeliac disease autoantibodies screening, sweat test, and RAST test to cow milk proteins were all negative. The diarrhea resolved under symptomatic treatment with exclusion of cow milk and did not relapse. Interestingly, during this episode, biological investigations revealed hemolytic anemia with positive direct Coombs test (AIHA). The patient received oral prednisone at 2 mg/kg/day during 1 month followed by a progressive decrease during 3 months with a stabilization of the hemoglobin rate. At the age of 14 months, he presented with an Evans syndrome as revealed by epistaxis and purpura. The patient’s platelet level was very low with a normal platelet size, and his anemia was critical, requiring transfusion. He underwent corticosteroid treatment (prednisone, 2 mg/kg/day) and received intravenous immunoglobulins (1 g/kg) with a poor response. During hospitalization, he also experienced a resolutive episode of pneumonia. The clinical picture was further worsened by multiple relapses of Evans syndrome, which required full-dose corticosteroid (prednisone, 2 mg/kg) and two hospitalizations at age 18 months and 3 years (**Figure 1**). Following the initial period of recurrent corticosteroid-dependent cytopenias, he continued to receive full dose of corticosteroids, allowing full remission at the age of 4 years. Interestingly, corticosteroid has been decreased then stopped for 3 years. His follow-up at age 7 years showed a favorable outcome. His condition is now characterized by the persistence of chronic adenopathies with no histological signs of malignancy and skin xerosis. During this follow-up, no gastro-intestinal manifestations, no endocrinopathies, or other auto-immune manifestations were recorded with the exception of a recent episode of thrombocytopenia. His infectious course was characterized by the occurrence of recurrent episodes of otitis media.

## Timeline

The timeline is presented in **Figure 1**.

### Diagnostic Assessment

**Figure 2:**
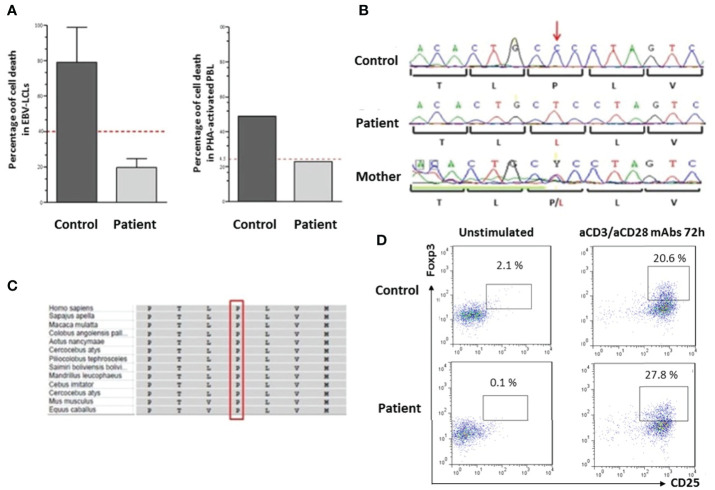
Genetic and functional investigations **(A)** Apoptosis assay: The IPEX patient exhibits a significantly altered apoptosis. Fas-induced apoptosis in EBV-lymphoblastoid cell lines (EBV-LCL) or PHA-activated peripheral blood lymphocytes of the patient and a healthy control were stimulated for 24 h with or without APO-1-3 in the presence of protein A. Subsequently apoptosis was measured by flow cytometric detection of Annexin V-PE and 7AAD. For apoptosis in EBV-LCL, data represent two separate experiments and the T bars indicate the standard deviation. The dotted red line represents 50% of the cell death observed in the control. **(B)** Genomic DNA sequence analysis of *FOXP3* gene showing a missens hemizygous mutation p.Pro75Leu in the patient, the mother was heterozygous for the mutation. **(C)** Multiple protein alignment showing the position of the conserved proline 75 residue in different species using the NCBI Multiple Sequence Alignment Viewer, Version 1.19.1. **(D)** FOXP3 expression in PBMCs after staining with anti-CD4/APC, anti-CD25/BB-515 and anti-Foxp3/PE as compared to healthy control. Treg cells were absent in patient’s unstimulated cells and induced after activation with anti-CD3 (aCD3)/anti-CD28 (aCD28) mAb during 72 h.

The diagnosis of IEI was suspected given the patient’s presentation. Preliminary immunological investigations showed polyclonal hypergammaglobulinemia (IgG: 15.51 g/L), elevated IgE, and hypereosinophilia. The standard immunophenotyping of lymphocyte subpopulations (T, B, and NK cells) assessed by flow cytometry was within the normal range. Lymphoproliferative responses to mitogens and antigens were normal. Recurrent Evans syndrome associated with chronic lymphoproliferations prompted the evaluation of ALPS criteria. Double negative T cells CD3+TCRα/β+CD4−CD8− percentage was increased, reaching 5.1%. Soluble FasL and IL-10 plasma levels were high. Apoptosis functional assay was performed on the patient’s EBV-transformed B-cells and showed severe resistance to Fas-mediated cell death in comparison to a healthy control, as confirmed in two separate experiments (**Figure 2A**). It has also been performed on the patient’s PHA-activated peripheral blood lymphocytes and confirmed the resistance to apoptosis as compared with healthy control (**Figure 2A**). This defect of apoptosis is persistent at age 7 years. All biological and immunological investigations are summarized in **Table 1**.

**Table 1:** Results of the immunological investigations.

Immunological Investigation	Results	Reference Values	Unit
Absolute leucocyte count	21.7	6.4–12.00	10^9^/L
Absolute lymphocyte count	7.1	3.60–8.90	10^9^/L
Absolute eosinophil count	9.8	<0.5	10^9^/L
**Flow cytometry lymphocyte phenotyping**			
T CD3+	91	53–75	% of Lc
T CD4+	52	32–51	% of Lc
T CD8+	25	14–30	% of Lc
TCR α/β+ CD4-CD8- (DNT cells)	**5­↑** ­	<1.5	% of CD3+ Lc
CD25+FOXP3+ (Treg cells)	**0.11↓**	–	% of CD4+ Lc
CD45RA+CCR7- (TEMRA cells)	**24↑ ­**	4–15	% of CD4+ Lc
**Lymphocyte proliferation tests**			
PHA	108307	Control: 98849	cpm
Anti-CD3	112209	Control: 80842	cpm
**Immunoglobulins**			
IgG	**15.51↑­**	3.4–6.2	g/L
IgA	1.12	0.33–1.22	g/L
IgM	**0.31↓**	0.48–1.43	g/L
IgE	**6046↑­**	<144	KU/l
**ALPS parameters**			
sFasL:	**1.4↑ ­**	≤0.2	ng/ml
IL-10 (tested twice):	**69.4↑ ­; 24↑ ­**	≤20	pg/ml
CD95:	67	–	% of Lc
Apoptosis functional assay in EBV-LCL:	**19.6↓**	Control: 79; 4	% of cell death
Apoptosis functional assay in PHA-PBL:	**23↓**	Control: 49	% of cell death
Vitamin B12 (tested twice):	1061; 564	345–1154	pg/ml
**Autoantibodies**			
Anti-nuclear antibodies	Negative	<1:80	Titer
Anti-DNA	Negative	<1:10	Titer
Anti-smooth muscle	Negative	<1:100	Titer
Anti-mitochondrial antibodies	Negative	<1:100	Titer
Anti-cardiolipin IgM	Negative	<12	PL U/ml
Anti-cardiolipin IgG	**20,81↑** ­	<12	PL U/ml
Anti-β2 glycoprotein 1 IgM	Negative	<5	PL U/ml
Anti-β2 glycoprotein 1 IgG	Negative	<5	PL U/ml
Rheumatoid factor	Negative	15	U/ml
Anti-cyclic citrullinated peptides	Negative	5	U/ml
Anti-TSH receptor	Negative	5	IU/ml
Anti- thyroperoxidase antibodies	Negative	<50	U/ml
Anti-intrinsic factor antibodies	Negative	–	NA
Autoantibodies against parietal cell antigens	Negative	–	NA
Anti-enzyme tissue transgulataminase IgA	Negative	<4	U/ml
Anti-deamidated gliadin peptide	Negative	<10	IU/ml
Anti-glutamic acid decarboxylase	Negative	<10	IU/ml

Altered parameters are in bold. ­: ↑­: increased; ↓: reduced. SI, Stimulation Index; NA, not applicable; DNT, cells: double negative t cells; Treg cells, T regulatory cells; TEMRA cells, terminally differentiated effector memory cells reexpressing CD45RA; PHA, phytohemagglutinin. Lymphocyte proliferation, expressed as radioactive counts per minutes (cpm) measured by 3H thymidine incorporation into DNA from the patient and a healthy control studied the same day. EBV-LCL, EBV-lymphoblastoid cell lines; PHA-PBL, PHA-activated peripheral blood lymphocytes; anti-TSH receptor, anti-thyroid stimulating hormone receptor.

The criteria for definitive ALPS diagnosis were fulfilled. Indeed, the two required criteria (i.e., expanded double negative T cells and chronic lymphadenopathy) were present together with one accessory primary criterion, namely, defective lymphocyte apoptosis in two separate assays and two accessory secondary criteria, namely, autoimmune cytopenia with elevated polyclonal IgG and elevated plasma sFasL levels (>200 pg/ml) and IL-10 levels (>20 pg/ml). The resistance to Fas-driven apoptosis argued in favor of a defect in the Fas pathway. The *FAS* gene was screened by Sanger sequencing for mutations in all exons and intron-exon junctions, because FAS deficiency accounts for approximately 65% of all ALPS cases. Surprisingly, no *FAS* germline mutation was found. Somatic *FAS* mutations were excluded because their presence does not fit with the defective apoptosis assay.

Targeted NGS, using the PID v2 gene panel (including all known ALPS genes) and an Ion Torrent S5 sequencer identified a novel missense mutation c.224C>T (p.Pro75Leu) located in *FOXP3*. No variants in other genes were found, which might have contributed to the patient’s phenotype. Sanger sequencing confirmed the mutation using forward and reverse specific primers (5′CCATGAGCCTCAGTTTCCAT and 5′ CACCTTTGACCCCCAGAGTA). The patient’s mother was heterozygous for the same mutation (**Figure 2B**). This mutation is located in the repressor domain of FOXP3 and is not reported in 1000G or ExAC databases. The affected residue is conserved among vertebrates (**Figure 2C**), and the mutation is predicted to be deleterious in multiple *in silico* prediction tools (probably damaging in Polyphen2, damaging in SIFT and CADD score of 24).

To assess the consequences of the *FOXP3* mutation, FOXP3 expression was performed in patient’s PBMCs as compared to healthy control, after staining with anti-CD4/APC, anti-CD25/BB-515 and anti-Foxp3/PE with or without activation for 72 h using anti-CD3/anti-CD28 monoclonal antibodies. FOXP3+ CD25+ cells were absent in patient’s unstimulated cells (0.1% of CD4+) but were induced after activation (27.8%) (**Figure 2D**).

The search for the presence of autoantibodies using an extensive set of antigens displayed negative results except for anti-cardiolipin IgG (**Table 1**).

**Figure 3:**
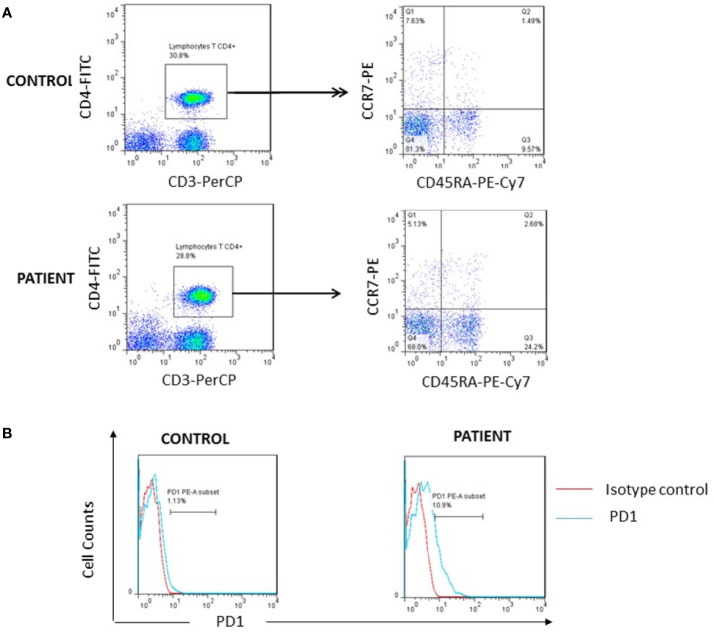
Investigation of T cell exhaustion. **(A)** High percentage of TEMRA cells (Q3) was found in patient after whole blood staining with anti-CD3/PerCP, anti-CD4/FITC, anti-CD45RA/PE-Cy7 and anti-CCR7/PE as compared to one representative healthy control. **(B)** Increased surface expression of PD1 on the patient’s CD4+ lymphocytes as compared to one representative control..

To decipher the potential link between FOXP3 defect and the ALPS phenotype, and based on the hypothesis that Treg deficiency would induce an unrestrained activation of T effector cells that are resistant to Fas driven apoptosis (19), we performed an immunophenotyping of TEMRA cells. These cells have been defined as the latest stage of T-cell differentiation which accumulate through successive rounds of antigen encounters (20, 21) and could be induced by persistent re-stimulation of individual clones (22). We found increased percentage of CD4+ TEMRA cells in the patient reaching 24% (normal range for the age, 4–15%) (**Table 1** and **Figure 3A**). Moreover, the patient showed high PD1 expression by 10.9% on CD4+T cells, one of the hallmarks of T cell exhaustion (23), in comparison to healthy controls (**Figure 3B**).

## Discussion

The classical IPEX presentation consists of a triad of symptoms, comprising diarrhea, autoimmune endocrinopathies and dermatitis (5, 24, 25). The most consistent clinical finding, often inaugural in this disease, is the exudative enteropathy (24). It classically begins very early, during the first few months of life, characterized by its persistence, and usually complicates with malabsorption and failure to thrive (5, 24, 25). The patient we report did first present with dermatitis and multiple adenopathies. Unlike in the IPEX classical presentation, this patient had a single limited episode of severe diarrhea at age ten months. Shortly after, he developed Evans syndrome associated with bleeding complications. Autoimmune cytopenias are a characteristic feature of ALPS. However, they are also observed in up to 26% of IPEX patients (25, 26). Moreover, the patient did not develop autoimmune endocrinopathies, which are frequently reported in IPEX patients and consist most commonly in type 1 diabetes mellitus occurring in the first year of life (5, 24, 25). It has been reported that in the absence of clinical disease, anti-glutamic acid decarboxylase antibodies or anti-islet cell antibodies may be positive (5). The screening of this patient’s serum for autoantibodies associated with endocrinopathies was negative, and no endocrine disorders were recorded during the follow-up. The patient’s clinical presentation was also marked by the occurrence of infectious manifestations consisting of recurrent media otitis and one episode of resolving pneumonia. This contrasts with the classical severe infections observed in IPEX patients, which may be life-threatening sepsis (5, 24, 25). Thus, the IPEX clinical presentation in this patient is unusual with mild or absent features of the characteristic IPEX triad and the predominance of an Evans syndrome. In this context, the persistence of benign adenopathies and hypergamma- globulinemia shaped a clinical picture more reminiscent of ALPS, the most typical IEI associated with autoimmune cytopenias (27). Moreover, the immunohistological pattern of sinus histiocytosis with S100 positive cells observed in the patient’s lymph node biopsy has not only been reported in ALPS (6) but also in a single description of IPEX patient (28).

The patient’s laboratory investigations showed hyper- eosinophilia and elevated IgE. The increase of these two parameters is common in IPEX patients. Although they are not usually characteristic of ALPS classical presentation, their increase was reported in patients with ALPS (29–31). Besides these overlapping features, the most striking finding in this observation is the presence of almost all ALPS biomarkers.

To our best knowledge, there are no published reports of IPEX associated with elevated ALPS biomarkers and a defective Fas apoptosis assay. Elevated DNT cells were initially considered as a hallmark of ALPS (9) and are still a required criterion for the diagnosis (12). This biomarker specificity has been challenged because other IEIs have displayed elevated DNT cells, such as LRBA deficiency, STAT3 GOF disease and CVID (13, 16). Elevation of DNT cells has also been reported in patients with autoimmune diseases (8, 32). However, when they exceed 4%, they are considered as highly predictive of *FAS* mutations (11). Nevertheless, high percentages of this subset, exceeding this threshold, have been recorded in LRBA deficiency, reaching 10% and in *STAT3* GOF disease reaching 20% (13, 16). In our patient, DNT cells reached 5% and were associated with other ALPS biomarkers, including elevation of IL-10 and sFasL and a defective Fas-induced apoptosis. Interestingly, in a recent review of key diagnostic markers of ALPS, the combination of elevated DNTs and an abnormal *in vitro* apoptosis functional test was considered the most useful in identifying all types of ALPS patients (33). The combination of an abnormal *in vitro* apoptosis functional test and elevated sFasL was a predictive marker for ALPS-FAS group classification (33).

The *FOXP3* mutation identified changed the diagnosis from ALPS to IPEX, clearly showing that NGS use is critical to unmask atypical presentations of IPEX and is reshaping the clinical and immunological spectrum of this rare and probably under-diagnosed disease (34). No less than 70 pathological variants have been identified in the *FOXP3* gene (5, 35). Interestingly, some mutations were more frequently associated with a mild or atypical presentation (34), such as reported here with currently a favorable outcome. This contrasts with the classical outcome of IPEX syndrome, generally associated with a poor prognosis (24). The mutation we report resides in the N terminal proline-rich domain, thought to mediate the repressive activity of FOXP3 (36). On another hand, the mutation is located in exon 3, which is the second coding exon (the first being a non-coding one) of a total of 12 exons. It is worth mentioning that some of these exons could be alternatively spliced leading to naturally occurring FOXP3 isoforms and may be associated with slower kinetics of FOXP3 export (37, 38). The mutation we identified was associated with the absence of CD4+CD25+FOXP3+ cells. Since steroid administration may reduce FOXP3 expression, the latter was assessed after activation and found to be restored. This finding is in accordance with the study of Gambineri et al. (39), showing that some FOXP3 mutations may not hinder protein expression after activation despite initial very low levels of Treg. The assessment of the correlation between restored FOXP3 expression and residual Treg function must be further investigated and may explain the moderate clinical phenotype of the patient.

The defect of apoptosis assay observed in the patient is somewhat intriguing. An altered functional apoptosis assay was previously observed in other immune regulation disorders, such as STAT3 GOF disease and LRBA deficiency (14, 16). Resistance to apoptosis in STAT3 GOF disease appears to be secondary to a disturbance in the balance of BCL2 family proteins, which are critical intrinsic regulators of the apoptotic pathway (16). The compromised Fas triggered apoptosis described in LRBA deficiency is suggested to be due to an altered autophagy. Indeed, autophagy is significantly reduced in LRBA deficient cells and has been shown to facilitate Fas-mediated apoptosis (14).

The Treg deficiency in the patient could result in unrestrained activation of T effector cells. The latter would become exhausted and thus resistant to apoptosis (19). Consistent with this hypothesis, we found increased CD4+TEMRA cells and PD1 expression in patient as compared with healthy controls.

On the other hand, defective FOXP3 observed in the patient could intrinsically induce increased proliferation and resistance to apoptosis in T effector cells, as herein reported. There is evidence in the literature that the amount of FOXP3 expressed within a cell controls the balance between life and death. Indeed, it has been shown that T cells from mice and humans that lack functional FOXP3 are hyper-responsive to TCR stimulation, and those from mice that overexpress FOXP3 are hypo-responsive to TCR-mediated stimulation as manifested by the reduction in proliferative capacity. Consequently, defect in cell cycle progression after TCR engagement may result in altered apoptosis (40).

In summary, this case expands the clinical spectrum of Foxp3 deficiency and suggests a potential link between FOXP3 and the FAS apoptotic pathway that requires further investigation.

## Patient Perspective

The patient did adhere to the treatment proposed. He and his family were satisfied by the relative improvement of his clinical condition.

## Data Availability Statement

The datasets presented in this study can be found in online repositories. The names of the repository/repositories and accession number(s) can be found below: https://www.ncbi.nlm.nih.gov/clinvar/variation/1098427/.

## Ethics Statement

The studies involving human participants were reviewed and approved by Comité d’éthique biomédicale de l’Institut Pasteur de Tunis. Written informed consent to participate in this study was provided by the participants’ legal guardian/next of kin.

## Author Contributions

AR, NM, NA and IB-M performed immunological investigations and interpretation of results. MS performed the screening of autoantibodies and interpretation of results. FF and MB were the clinicians in charge of patient care and management. MFA and RG performed targeted NGS and interpretation of results. IB-M, M-RB, and RG contributed to conception and design of the study. AR and IB-M wrote the initial manuscript draft. AR, NM, MA, RG, IB-M and M-RB reviewed the manuscript and contributed to the final draft. All authors contributed to the article and approved the submitted version.

## Funding

This work was supported by the Tunisian ministry of higher education and research.

## Conflict of Interest

The authors declare that the research was conducted in the absence of any commercial or financial relationships that could be construed as a potential conflict of interest.

## Publisher’s Note

All claims expressed in this article are solely those of the authors and do not necessarily represent those of their affiliated organizations, or those of the publisher, the editors and the reviewers. Any product that may be evaluated in this article, or claim that may be made by its manufacturer, is not guaranteed or endorsed by the publisher.
